# Supplementary material for: Dynamic nucleosome landscape elicits a noncanonical GATA2 pioneer model
Source: Nat Commun. 2022 Jun 7;13:3145. doi: 10.1038/s41467-022-30960-x (PMC9174260; doi:10.1038/s41467-022-30960-x)
Supplement: Supplementary file 1 — Supplementary Information [file 41467_2022_30960_MOESM1_ESM.pdf]

# Supplementary Information

## Dynamic nucleosome landscape elicits a novel noncanonical GATA2 pioneer model

Tianbao Li, Qi Liu, Zhong Chen, Kun Fang, Furong Huang, Xueqi Fu, Qianben Wang, Victor Jin

## Supplementary Figures

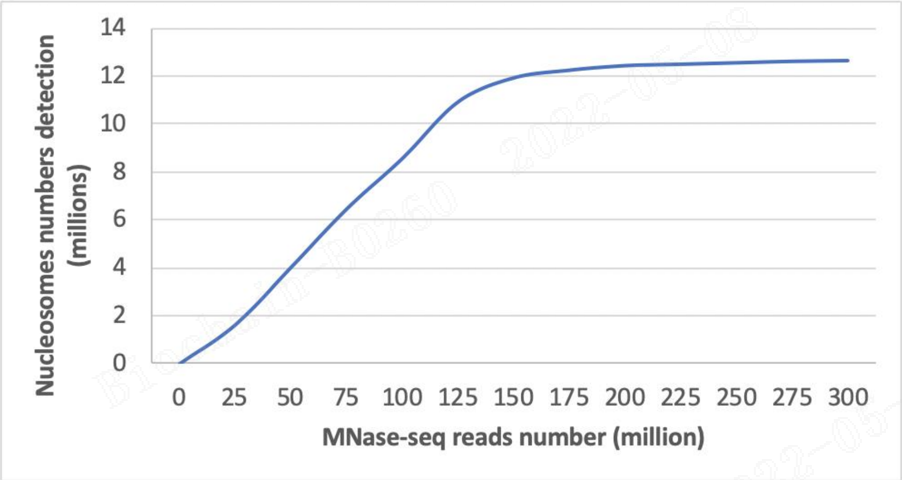


Supplementary Figure 1. Genome-wide nucleosomes detection curve of MNase-seq datasets. A total of 12 sets of different sequencing depth of raw reads (25 to 300 millions) were applied to detect the saturation number of nucleosomes.

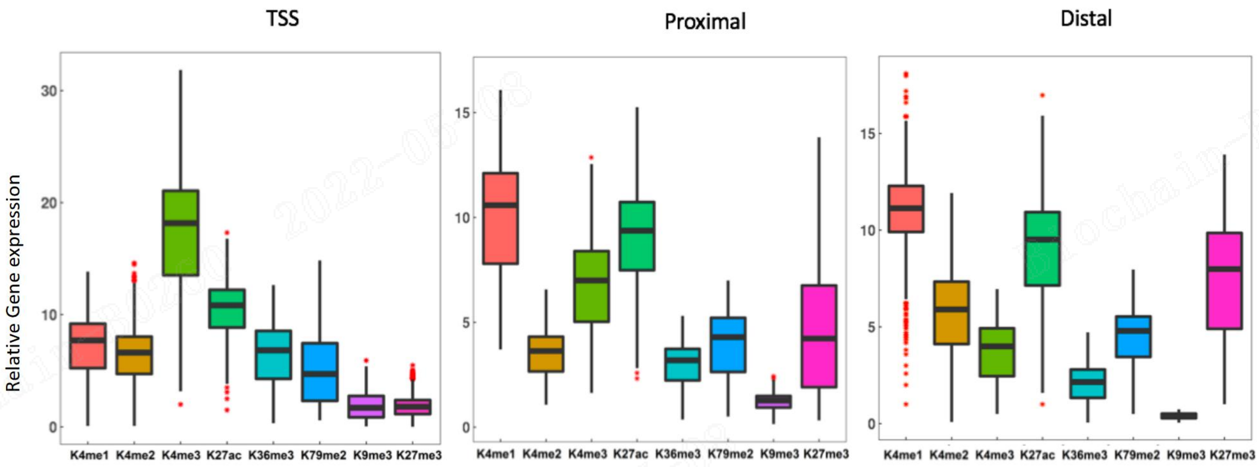


Supplementary Figure 2. Relative gene expression associated with various histone marks in different genomic regions with n=2 biologically independent samples. Eight histone marks in TSS, Proximal and Distal regions were compared with adjacent relative gene expression. The lines in the boxes present the medians of results. The lower and upper bounds of the boxes indicate 25th and 75th percentiles.

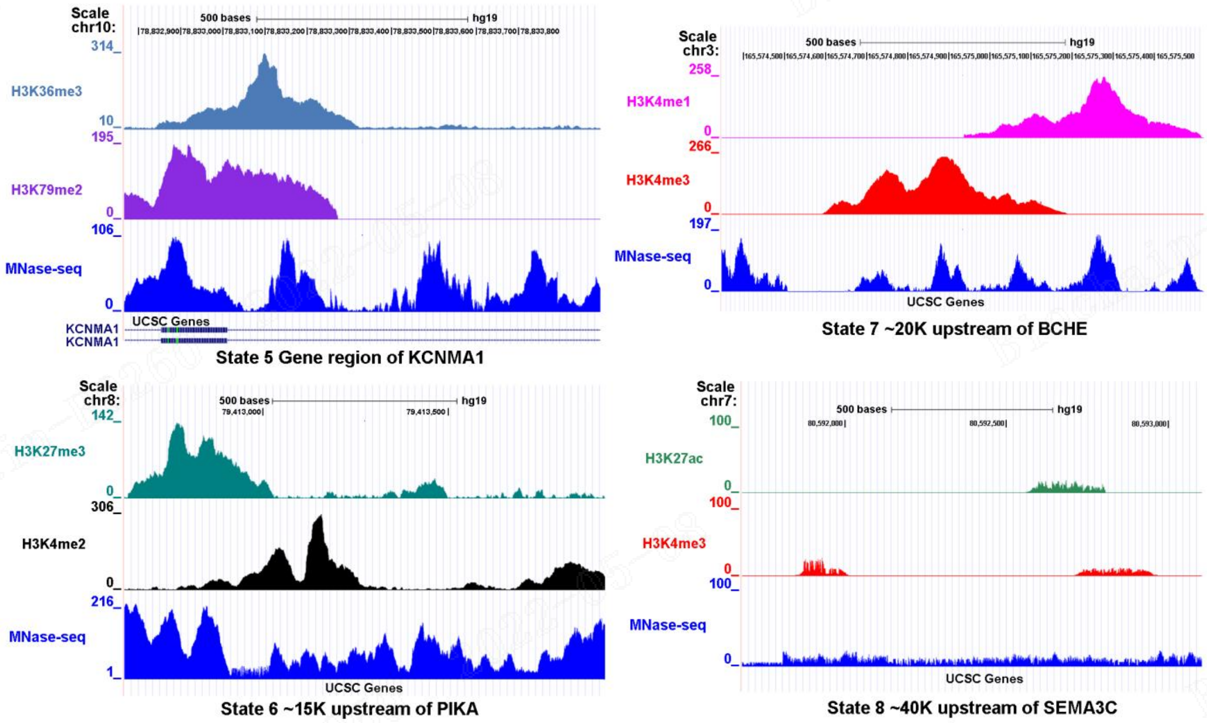


Supplementary Figure 3. The visualization of functional nucleosome states: S5 (up-left), S6 (down-left), S7 (up-right) and S8 (down-right).

Biochain-B0260 2022-05-08

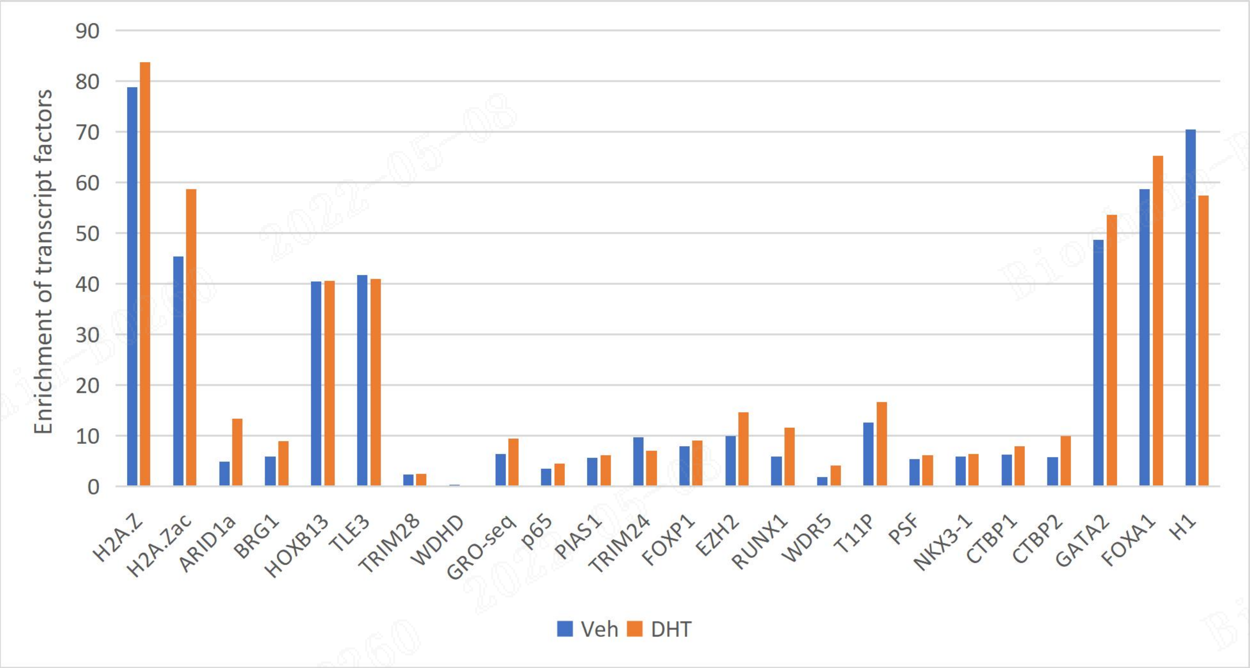


Supplementary Figure 4. The enrichment of transcript factors and histone marks under Veh and DHT LNCaP conditions. Each transcript factor was detected in the nucleosome states location and accumulated as an overall enrichment.

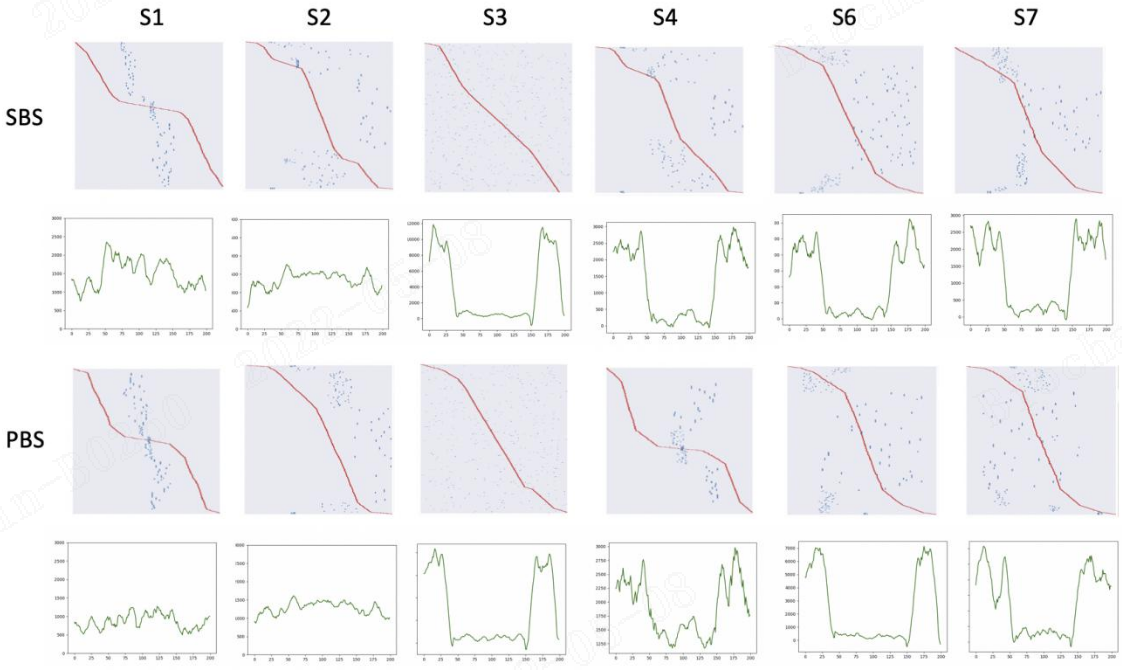


Supplementary Figure 5. The enrichment of H1 within FOXA1 borders. Single Border Site (SBS) and Paired Border Site (PBS) of FOXA1 ChIP-exo dataset were detected in different nucleosome states (upper panel), and the enrichment of H1 in the same loci was showed in the bottom panel.

Biochain-B0260 2022-05-08

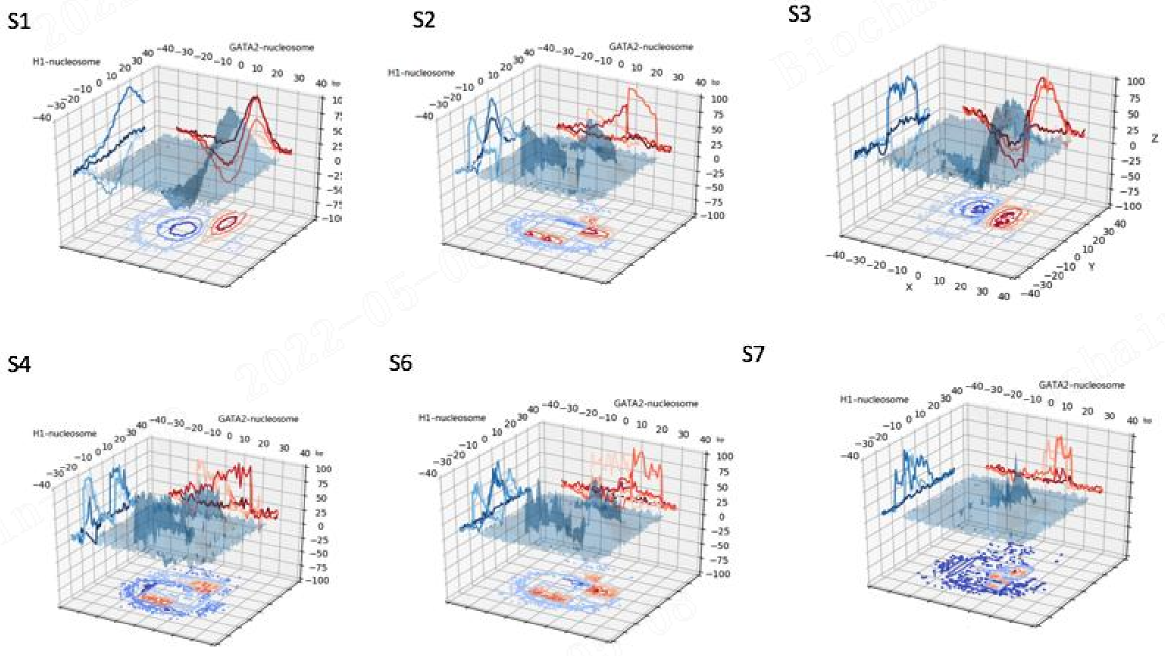


Supplementary Figure 6. An association between H1 and GATA2 enrichment in different nucleosome states. The contour plot showed the co-location around nucleosome dyad of H1-occupied and GATA2-occupied nucleosomes.

Biochain-B0260 2022-05-08

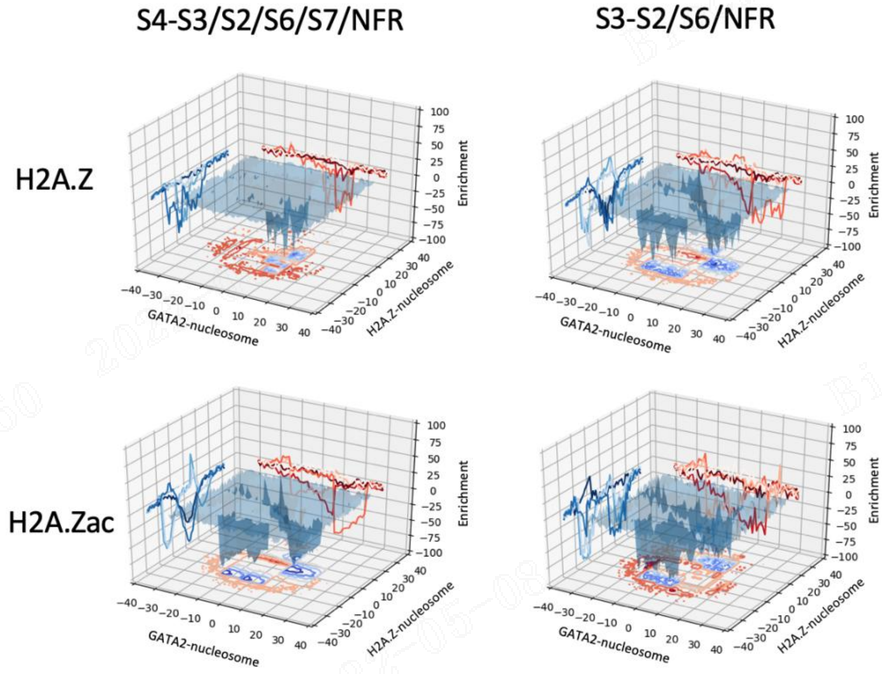


Supplementary Figure 7. The enrichment of H2A.Z and H2A.Zac accompanying with GATA2-associated nucleosome states switches. H2A.Z modification was decreased with GATA2 binding in S4-S3/S2/S6/S7/NFR states, while H2A.Zac showed higher connection with GATA2 binding in S3-S2/S6/NFR states.

Biochain-B0260 2022-05-08

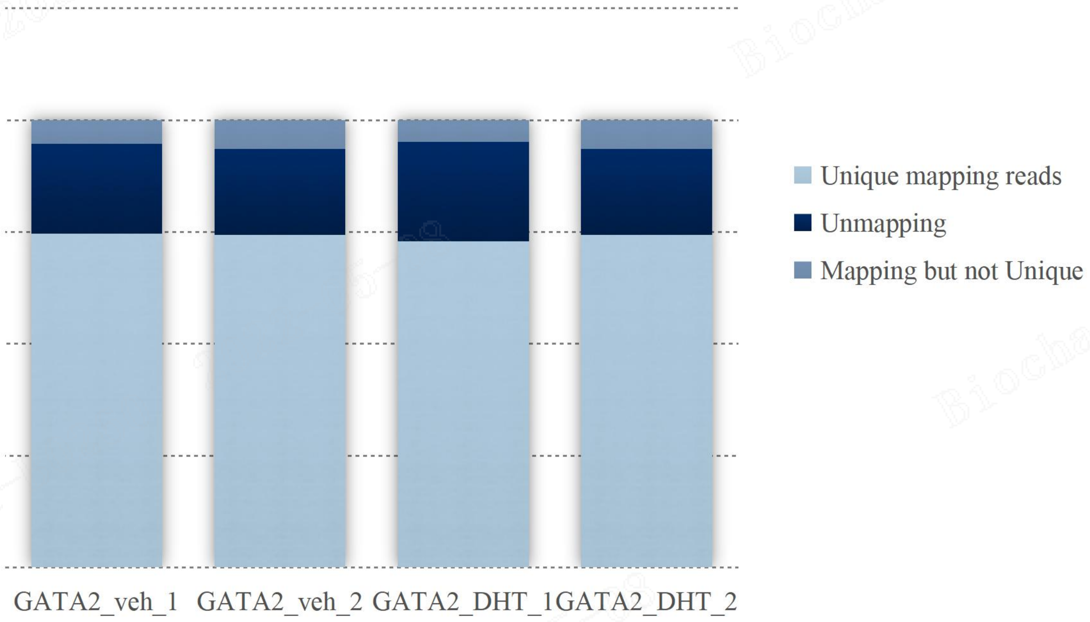


Supplementary Figure 8. The Mapping quality between replicates of GATA2 ChIP-ePEST and the Pearson correlation of raw reads count within 200bp bin size are r>0.95 for both veh and DHT data between replicates.

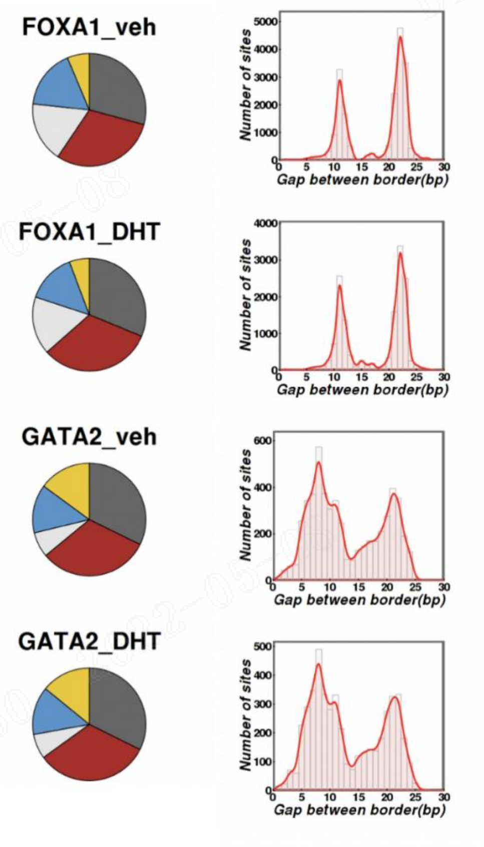


Supplementary Figure 9. A comparison of FOXA1 and GATA2 borders showing both display the bimodal distribution. The distribution of FOXA1 and GATA2 border types (left) and borders gap between paired border sites.

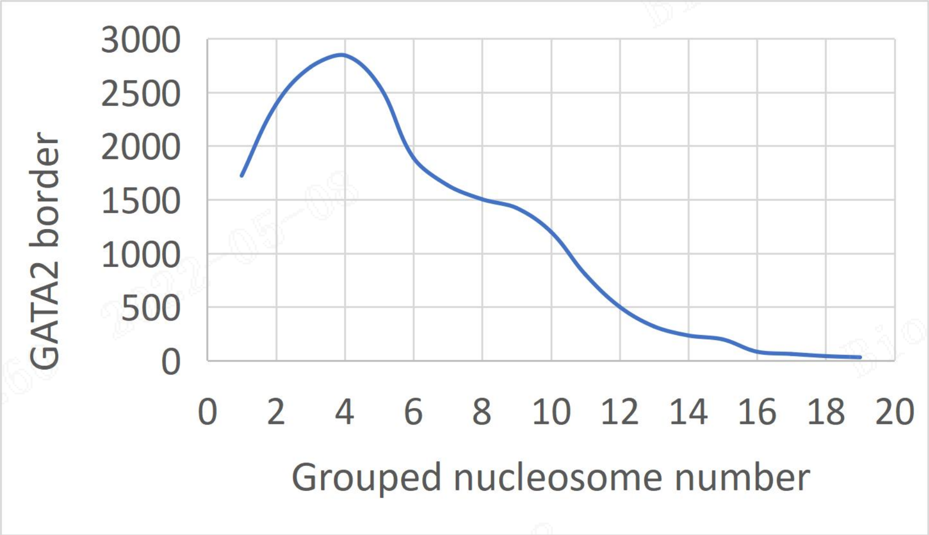


Supplementary Figure 10. The relationship of GATA2 border binding with grouped nucleosomes. GATA2 borders were accumulated by different nucleosome numbers.

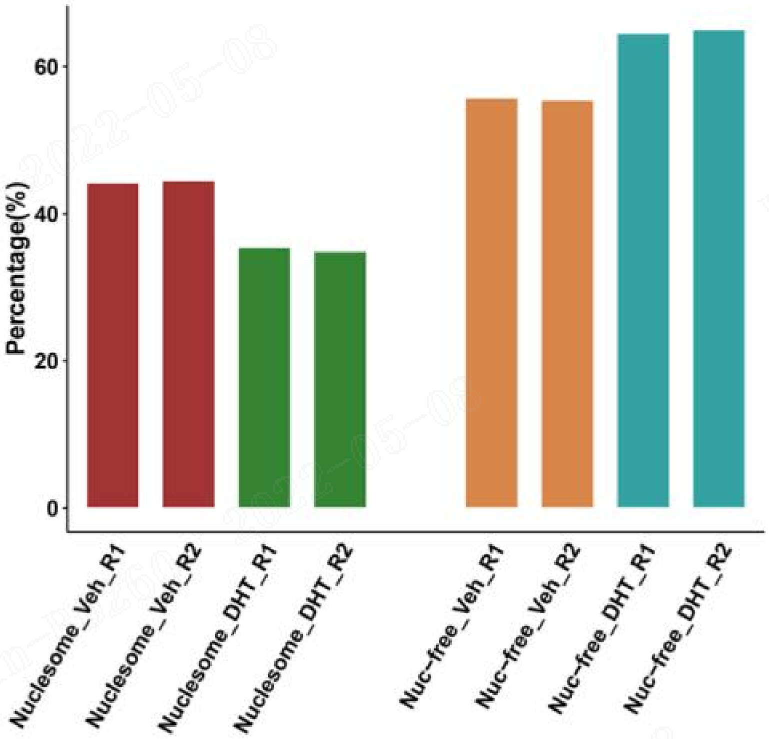


Supplementary Figure 11. GATA2 border binding in the nucleosomes region in DHT treated LNCaP cells.

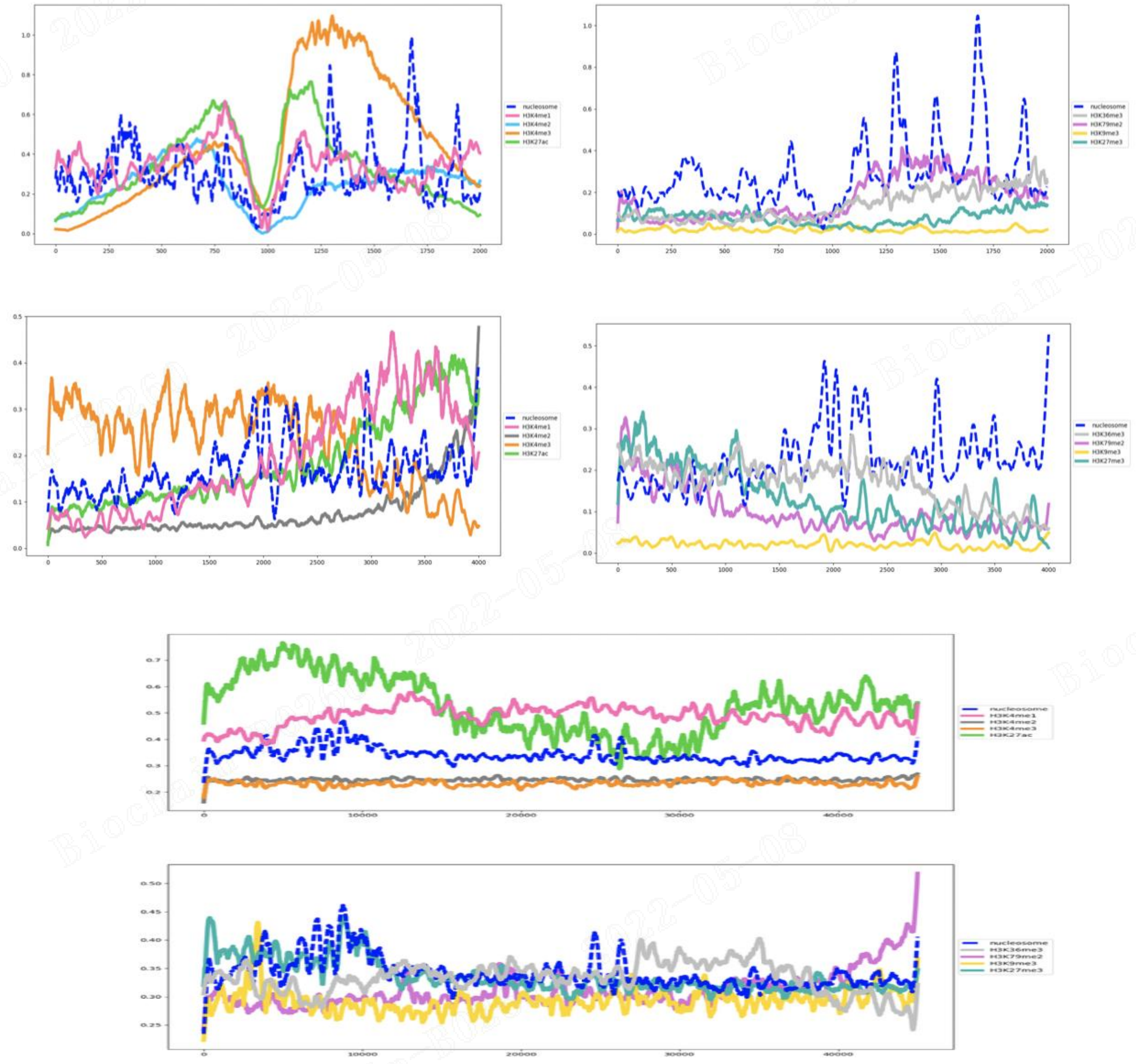


Supplementary Figure 12. Nucleosome and histone marks enrichment in three genomic regions with DHT treatment condition in LNCaP cells. TSS region (upper), Proximal (middle) and Distal region (bottom) were shown in the different colors.

Biochain-B0260 2022-05-08

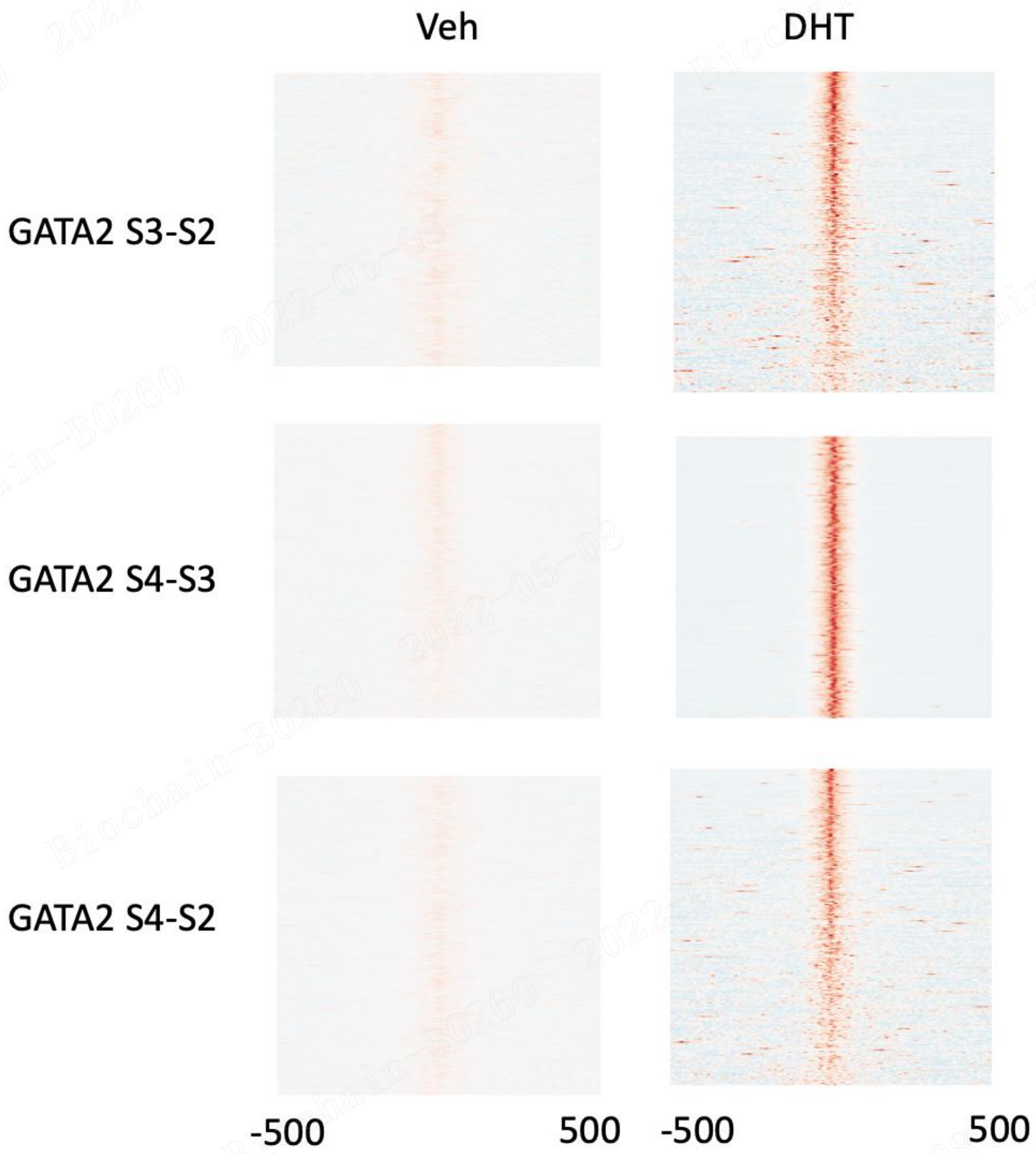


Supplementary Figure 13. GATA2 border enhanced within DHT treated LNCaP cells. The GATA2 binding bases were set as 0 and nucleosomes in each line were sorted by the highest MNase-seq raw reads.

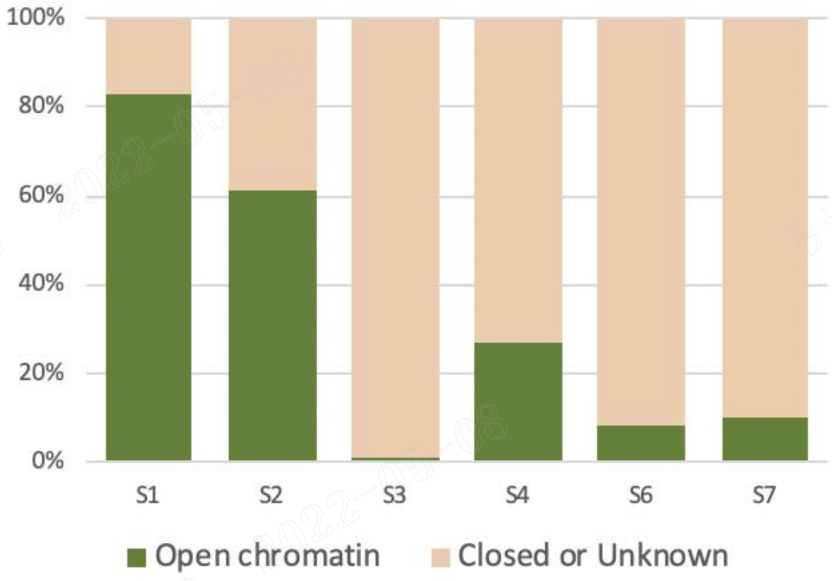


Supplementary Figure 14. Open chromatin measured by ATAC-seq in various nucleosome states. The ATAC-seq reads were detected within the 50bp of upstream/downstream nucleosome state regions.

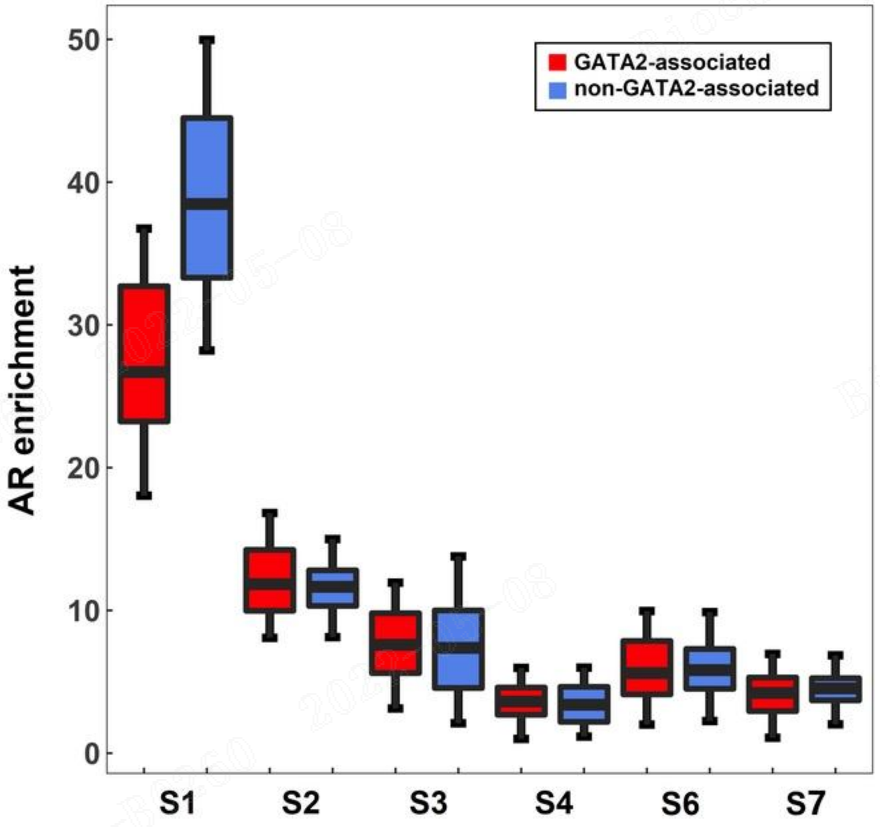


Supplementary Figure 15. AR enrichment in GATA2-associated (left) and non-GATA2-associated (right) nucleosome states with n=2 biologically independent samples. AR enrichment showed no significant difference between GATA2 associated state and non-associated states, except in state S1. The lines in the boxes present the medians of results. The lower and upper bounds of the boxes indicate 25th and 75th percentiles.

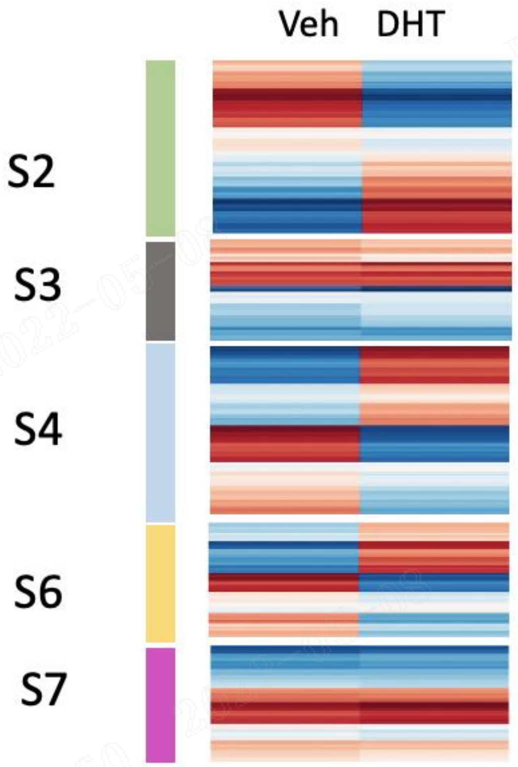


Supplementary Figure 16. Gene expression changes associated with different nucleosome states upon DHT-treated LNCaP cells. Genes associated with changed nucleosome states in the DHT treatment condition were analysed for differential gene expression.

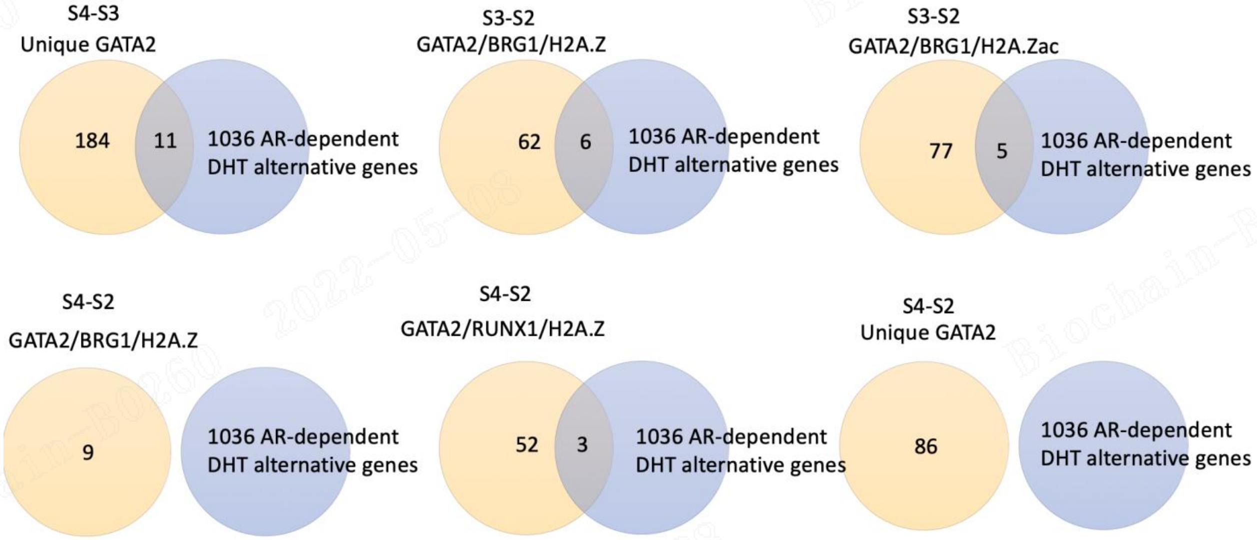


Supplementary Figure 17. Venn diagrams showing overlaps among GATA2-associated nucleosome states switches, other co-regulators and AR-dependent DHT-treated DEGs. The overlapping results were based on genes associated with switched nucleosome states.

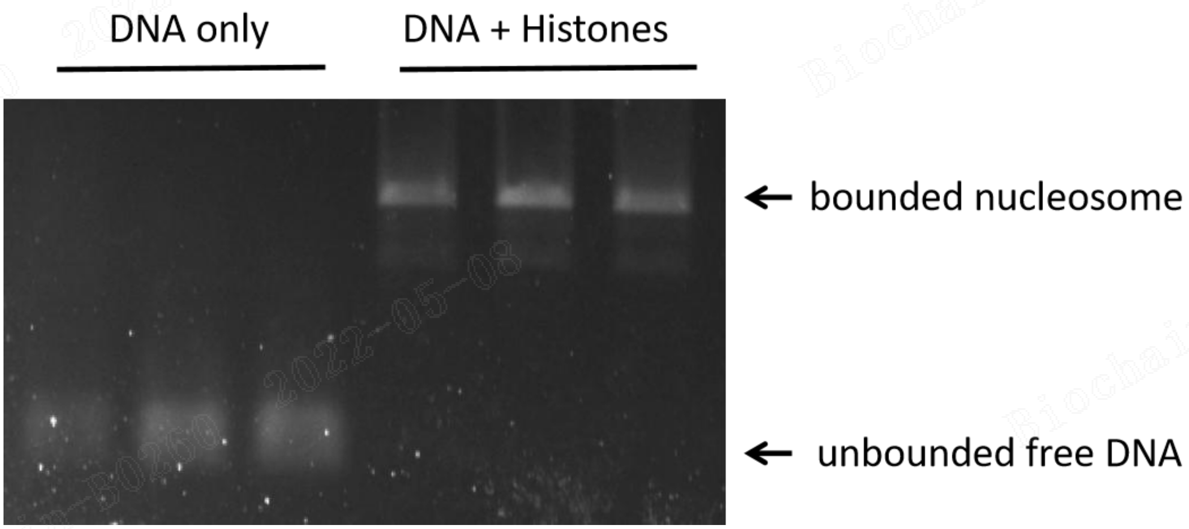


Supplementary Figure 18. In vitro nucleosomes generation detection. H2A/H2B dimer and H3.1/H4 tetramer were mixed with synthesized double-stranded DNA with GATA2 motif.

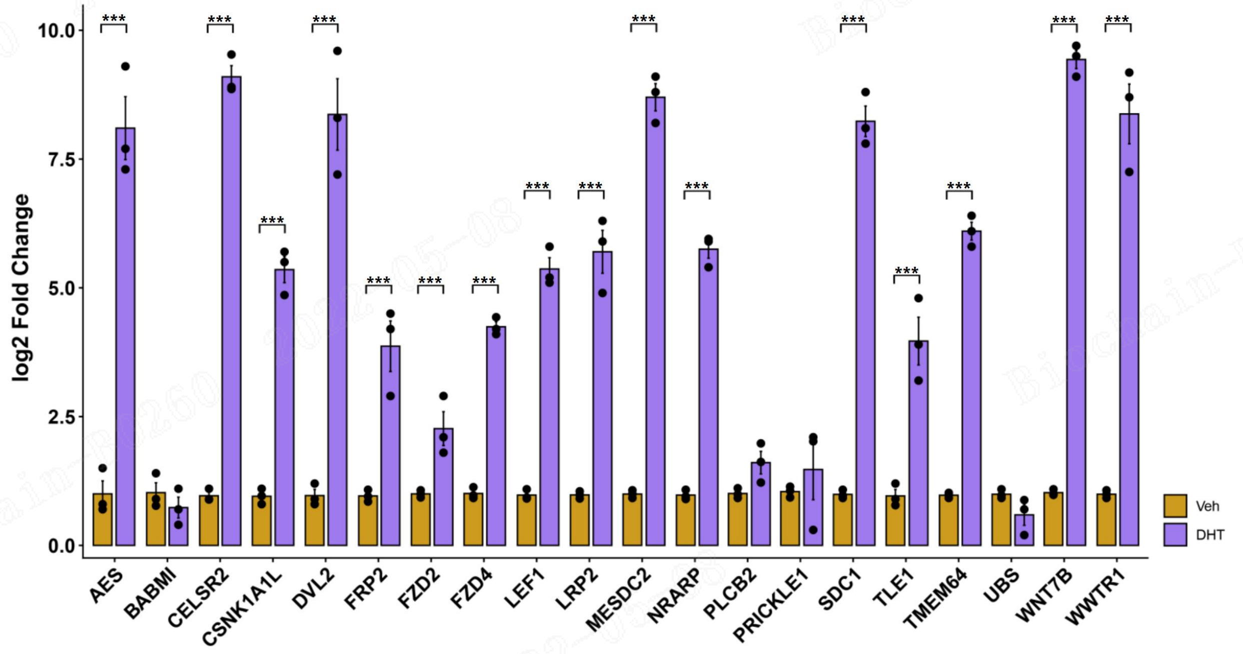


Supplementary Figure 19. Open chromatin assay is conducted to identify differential chromatin openness between DHT treated and Veh conditions. We demonstrated that 17 of 20 Wnt/β-catenin signaling genes showed an increase of chromatin opening upon DHT treatment. The statistical analysis of qPCR results were performed by t-test, while the "***" present the p-value < 0.001 (two-tailed unpaired Student's t test) and the "*" present the p-value < 0.05 between Veh and DHT groups of each gene loci. Each bar represents the mean value with the standard deviation as error bars. Source data are provided as a Source Data file

Biochain-B0260 2022-05-08

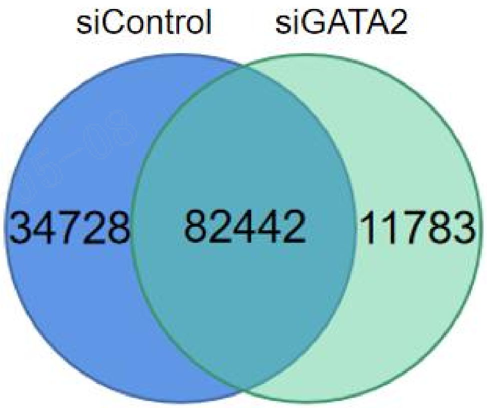


Supplementary Figure 20. The genome-wide ATAC-seq data analysis for siGATA2 and siControl conditions in LNCaP cell line.

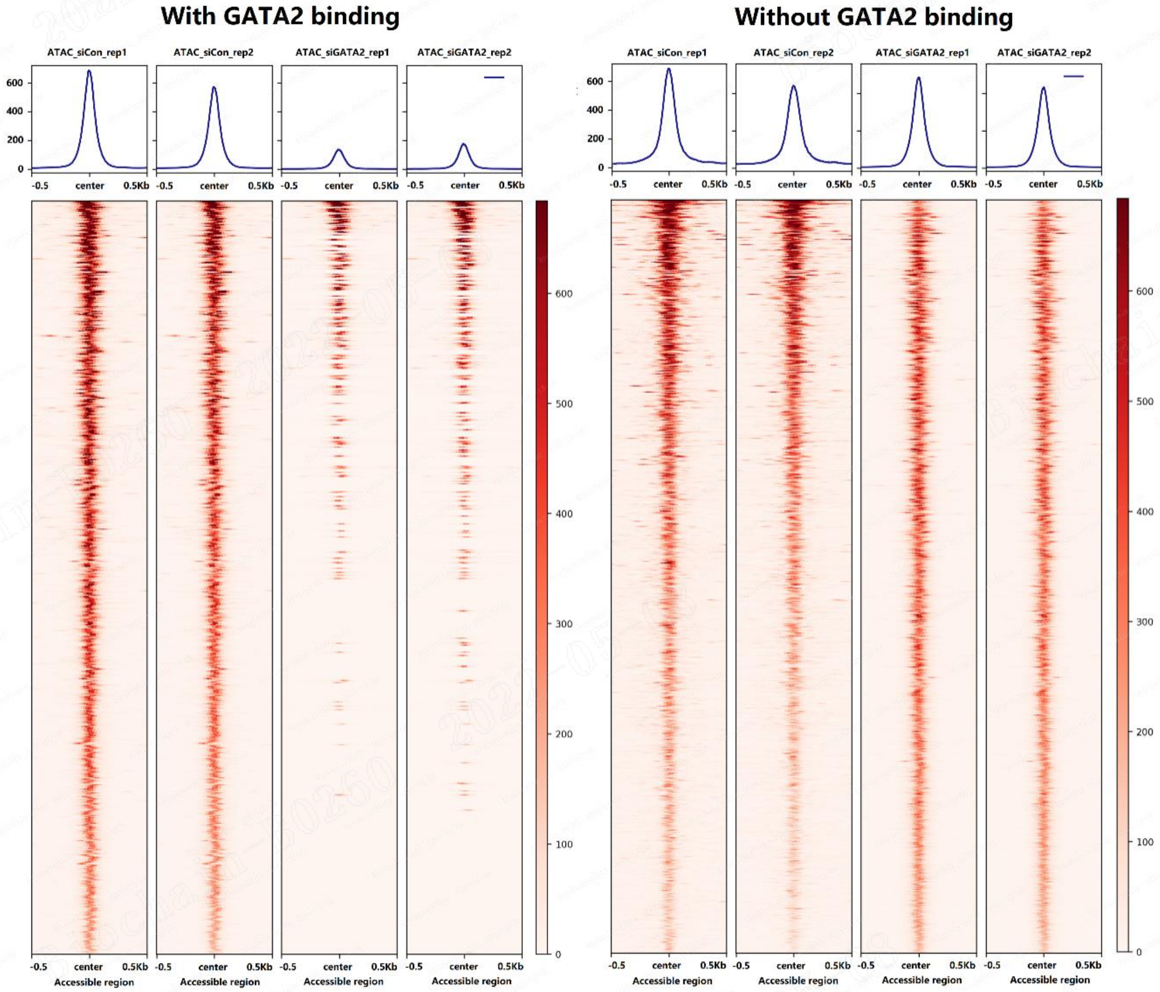


Supplementary Figure 21. ATAC-seq reads density maps with GATA2 binding sites (left) and without GATA2 binding sites (right) within 0.5Kb up/down-stream in siCtrl LNCaP cell line and siGATA2 subline. The data demonstrated that GATA2-associated binding regions significantly reduced the chromatin accessibility.

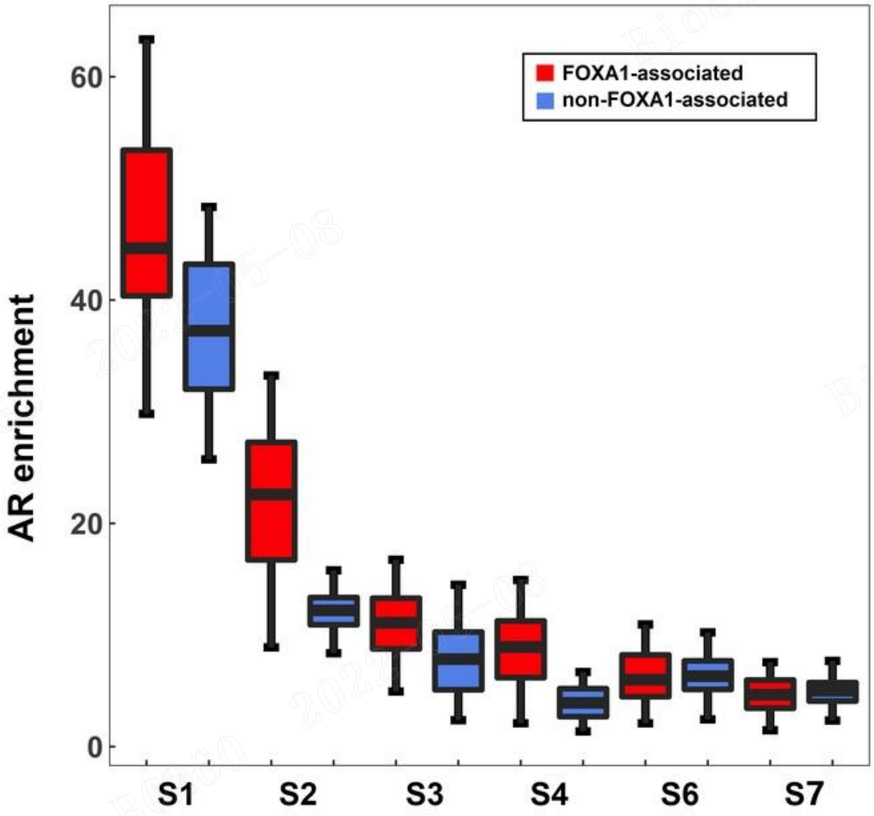


Supplementary Figure 22. AR enrichment in FOXA1-associated (left) and non-FOXA1-associated (right) nucleosome states. AR enrichment showed the difference between FOXA1 associated states and non-associated states with n=2 biologically independent samples. The lines in the boxes present the medians of results. The lower and upper bounds of the boxes indicate 25th and 75th percentiles.

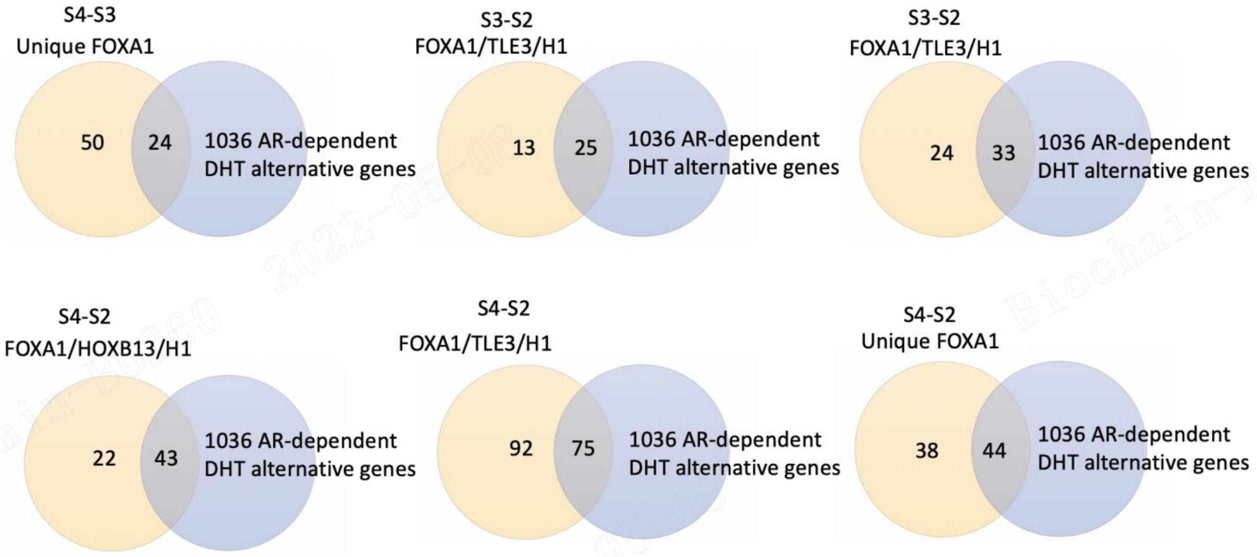


Supplementary Figure 23. Venn diagrams showing overlaps among FOXA1-associated nucleosome states switches, other co-regulators and AR-dependent DHT-treated DEGs. The overlapping results were based on genes associated with switched nucleosome states.

# Supplementary Tables

Supplementary Table S1. A summary of raw sequencing reads in LNCaP cells.

| Type | Antibody | Veh (Rep 1/Rep 2) | DHT (Rep 1/Rep 2) |
|---|---|---|---|
| MNase-seq | mono-nucluesome | 361M / 354M | 366M / 341M |
| GATA2-ChIP-exo | GATA2 | 194M / 171M | 190M / 170M |
| MNase-ChIP-seq | H3K4me1 | 36.4M / 34.3M | 37.2M / 35.3M |
| | H3K4me3 | 36.6M / 34.1M | 35.2M / 35.7M |
| | H3K27ac | 45.3M / 51.1M | 39.2M / 42.3M |
| | H3K9me3 | 41.4M / 36.5M | 40.2M / 38.7M |
| | H3K27me3 | 36.5M / 41.6M | 38.6M / 42.3M |
| | H3K36me3 | 40.4M / 42.5M | 38.4M / 34.6M |
| | H3K79me2 | 35.2M / 36.5M | 34.3M / 35.2M |
| ChIP-seq | H1 antibody | 33.1 M / 31.5M | 30.2 M / 33.4M |
| Summary | 10 types | 1.63 billion for 20 samples | 1.65 billion for 20 samples |

M is short for a Million.



Biochain-B0260 2022-05-08

Supplementary Table S2. Nucleosome coverage in different chromosomes.

| | Nucleosome cover bp | Total bp | Coverage rate |
|---|---|---|---|
| Chr 1 | 121303355 | 249250621 | 48.7% |
| Chr 2 | 127413655 | 243199373 | 52.4% |
| Chr 3 | 108119135 | 198022430 | 54.6% |
| Chr 4 | 101079195 | 191154276 | 52.9% |
| Chr 5 | 96650265 | 180915260 | 53.4% |
| Chr 6 | 91221945 | 171115067 | 53.3% |
| Chr 7 | 82650675 | 159138663 | 51.9% |
| Chr 8 | 78600705 | 146364022 | 53.7% |
| Chr 9 | 60644805 | 141213431 | 42.9% |
| Chr 10 | 70938455 | 135534747 | 52.3% |
| Chr 11 | 71780535 | 135006516 | 53.2% |
| Chr 12 | 72203265 | 133851895 | 53.9% |
| Chr 13 | 49858555 | 115169878 | 43.3% |
| Chr 14 | 48631335 | 107349540 | 45.3% |
| Chr 15 | 43010815 | 102531392 | 41.9% |
| Chr 16 | 40626735 | 90354753 | 45.0% |
| Chr 17 | 41926965 | 81195210 | 51.6% |
| Chr 18 | 40555255 | 78077248 | 51.9% |
| Chr 19 | 29607085 | 59128983 | 50.1% |
| Chr 20 | 33596625 | 63025520 | 53.3% |
| Chr 21 | 18641945 | 48129895 | 38.7% |
| Chr 22 | 18069075 | 51304566 | 35.2% |
| Chr X | 71264585 | 155270560 | 45.9% |
| Chr Y | 5116245 | 59373566 | 8.6% |
| Total | 1,523,511,210 | 3,095,677,412 | 49.2% |



Biochain-B0260 2022-05-08

Supplementary Table S3. A summary of GATA2 ChIP-ePENS datasets.

| Datasets | GATA2_Veh1 | GATA2_Veh2 | GATA2_DHT1 | GATA2_DHT2 |
|---|---|---|---|---|
| Reads processed | 204,450K | 151,172K | 170,073K | 190.103K |
| Alignment reads | 163,371K | 122,181K | 132,326K | 153,648K |
| Unique mapping reads | 152,389K | 112,259K | 123,809K | 141,248K |

Supplementary Table S4. Public datasets used in this study

| No. | Transcript factor | GEO dataset | Corresponding Figure |
|---|---|---|---|
| 1 | H2A.Z | GSE76337 | Figure 3c, 3d, 3e |
| 2 | H2A.Zac | GSE27824 | Figure 3c, 3d, 3e |
| 3 | ARID1a | GSE94682 | Figure 3c, 3d, 3e |
| 4 | BRG1 | GSE94682 | Figure 3c, 3d, 3e |
| 5 | HOXB13 | GSE94682 | Figure 3c, 3d, 3e |
| 6 | TLE3 | GSE94682 | Figure 3c, 3d, 3e |
| 7 | TRIM28 | GSE94682 | Figure 3c, 3d, 3e |
| 8 | WDHD | GSE94682 | Figure 3c, 3d, 3e |
| 9 | TRIM24 | GSE69331 | Figure 3c, 3d, 3e |
| 10 | GRO-seq | GSE83860 | Figure 3c, 3d, 3e |
| 11 | p65 | GSE83860 | Figure 3c, 3d, 3e |
| 12 | PIAS1 | GSE83860 | Figure 3c, 3d, 3e |
| 13 | FOXP1 | GSE62492 | Figure 3c, 3d, 3e |
| 14 | EZH2 | GSE39459 | Figure 3c, 3d, 3e |
| 15 | RUNX1 | GSE39459 | Figure 3c, 3d, 3e |
| 16 | WDR5 | GSE55279 | Figure 3c, 3d, 3e |
| 17 | T11P | GSE55279 | Figure 3c, 3d, 3e |
| 18 | PSF | GSE45124 | Figure 3c, 3d, 3e |
| 19 | NKX3-1 | GSE28264 | Figure 3c, 3d, 3e |
| 20 | CTBP1 | GSE58428 | Figure 3c, 3d, 3e |
| 21 | CTBP2 | GSE58428 | Figure 3c, 3d, 3e |
| 22 | AR (ChIP-exo) | GSE143907 | Figure S15, S23 |
