# Supplementary material for: Dynamic nucleosome landscape elicits a noncanonical GATA2 pioneer model
Source: Nat Commun. 2022 Jun 7;13:3145. doi: 10.1038/s41467-022-30960-x (PMC9174260; doi:10.1038/s41467-022-30960-x)
Supplement: Supplementary file 3 — Description of additional Supplementary File [file 41467_2022_30960_MOESM3_ESM.pdf]

Descriptions of additional supplementary data files

Supplementary Data 1: List of GATA2 borders in Veh and DHT conditions.

Supplementary Data 2: List of GATA2 occupied nucleosomes.

Supplementary Data 3: List of S4/3 to RAS with GATA2 binding.
