# Supplementary figures and images for: Dynamic nucleosome landscape elicits a noncanonical GATA2 pioneer model
Source: Nat Commun. 2022 Jun 7;13:3145. doi: 10.1038/s41467-022-30960-x (PMC9174260; doi:10.1038/s41467-022-30960-x)

Unprocessed scan for Figure 6a left panel:


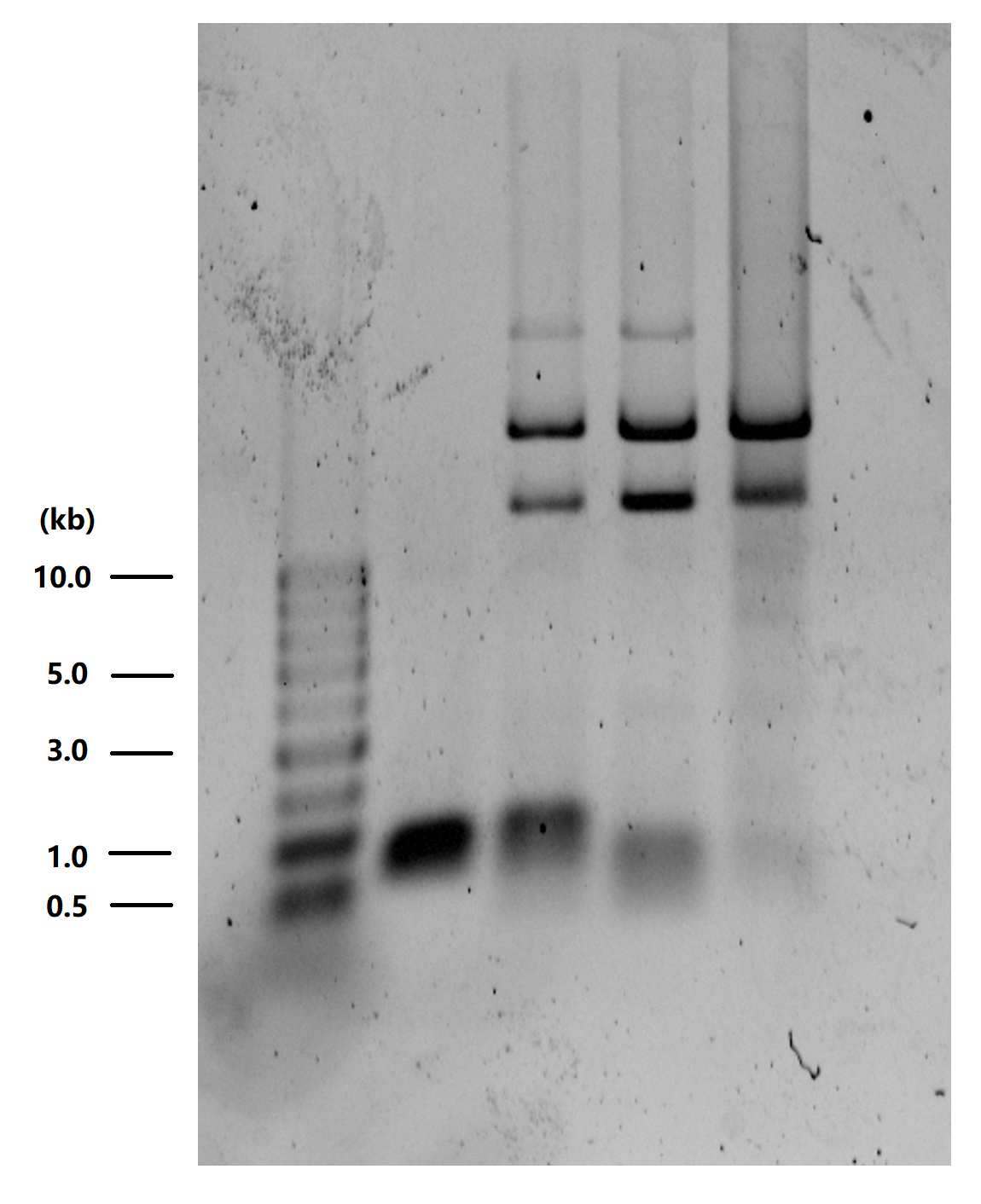

Supplement: Supplementary file 7 — Source Data [file 41467_2022_30960_MOESM7_ESM.zip › Source Data/Source Data 1_Figure 6a_uncropped western.docx]
